# A simple model for pink noise from amplitude modulations

**DOI:** 10.1038/s41598-023-34816-2

**Published:** 2023-05-24

**Authors:** Masahiro Morikawa, Akika Nakamichi

**Affiliations:** 1 Department of Physics, Ochanomizu University, 2-1-1, Otsuka, Bunkyo, Tokyo 112-8610 Japan; 2 General Education, Kyoto-Sangyo University, Motoyama Kamigamo, Kita-ku, Kyoto 603-8555 Japan

**Keywords:** Physics, Statistical physics, thermodynamics and nonlinear dynamics, Statistical physics

## Abstract

We propose a simple model for the origin of pink noise (or 1/f fluctuation) based on the waves with accumulating frequencies. These waves arise spontaneously in a system with synchronization, resonance, and infrared divergence. Many waves with accumulating frequencies can produce signals of arbitrary small frequencies from a system of small size. This beat mechanism can be understood as amplitude modulation. The pink noise can appear after the demodulation process, which produces a variety of pink noise in many fields. The pink noise thus formed from the beat has nothing to do with dissipation or long-time memory. We also suggest new ways of looking at pink noise in earthquakes, solar flares, and stellar activities.

## Introduction

Pink noise is ubiquitous. This noise is characterized by the power-law behavior in the very low-frequency region of the power spectrum density (PSD) with power $-\alpha$, ($0.5<\alpha <1.5$). This noise is also known as 1/f fluctuation or flicker noise.

Since the first discovery of pink noise in a vacuum tube current^1^, the same noise has been observed in many systems: semiconductors, thin metals, biomembranes, crystal oscillators, very long-term temperature variations, the loudness of orchestral music, fluctuations in the Earth’s rotation speed, fluctuations in the intensity of cosmic rays, heartbeats, postural control, magnetoencephalography, and electroencephalography in the brain, etc.^2,3^.

There have been many discussions about the origin of pink noise^2–5^, but there seems to be no clear conclusion. Many models have been proposed that give rise to pink noise, but no universal mechanisms have been discovered.

Since pink noise is ubiquitous, the mechanism should be simple enough. However, all the applications of the basic concepts and techniques of the standard statistical mechanics seem to have encountered conflicts and disputes. Then people have tended to consider more fundamental concepts that can rewrite the theory of standard statistical mechanics.

A typical mechanism for producing arbitrary low-frequency fluctuations would be the wave beat, or amplitude modulation, of the primary high-frequency fluctuations. This amplitude modulation would be successful for pink noise if the frequencies were more concentrated in a small range. Then the secondary beat wave can have lower frequencies. One of the authors has already proposed this mechanism for the pink noise of sounds and music^6^.

Furthermore, this concentration should be cooperative and systematic to form the power-law PSD. We propose at least three types of cooperative systems that can produce pink noise. They are (a) synchronization, (b) resonance, and (c) the infrared (IR) divergence.

If the pink noise were an amplitude modulation, the demodulation mechanism should also exist. This is because the entire modulated data has only high-frequency information, while the data after demodulation can explicitly show the low-frequency information, including the pink noise. The demodulation mechanism can be intrinsic to the system or be prepared in the measurement procedure. Many demodulation mechanisms make the pink noise phenomena diverse: taking the square of the original signal, rectification, thresholding, etc. For example, when the electrical current or voltage exceeds the threshold in the biological body, ignition occurs and produces spikes in the nerve cells. Thus the possible pink noise in the electric current is transferred to the nerve signal.

We begin our discussion in the next section Method, listing crucial clues to the origin of pink noise; all pointing to the possibility that pink noise is amplitude modulation. We then propose three mechanisms that lead to the modulation. We first discuss the most typical mechanism synchronization. We show that (a) exponential synchronization yields a power index of $-1,$ and power-law synchronization yields a power index slightly different from $-1$. Next, (b) resonance also yields pink noise since the concentration of the excited eigenmodes around the fiducial frequency is systematically approximated by the exponential function in the relevant domain. Further, (c) infrared divergence in the bremsstrahlung can give pink noise. Finally, we discuss the robustness of pink noise and several demodulation mechanisms that yield a variety of pink noise. In the conclusion section, we summarize our proposal and possible verifications based on the points presented in the Method section. We also summarize our prospects of amplitude modulation on a variety of systems.

## Method: some crucial clues for pink noise

We will now list some crucial clues to the origin of the pink noise. This process is quite important because it can clarify which principles of statistical mechanics are useful and which are not useful to describe the pink noise.

1. **Wave** Systems that exhibit pink noise are often waves: sound waves, electric current, air-fluid, liquid flow, etc. Waves can interfere with each other. Thus the interference of waves can be a clue to getting pink noise.
2. **Small system and seemingly long memory** It is bizarre that an ultra-low frequency signal can come from a tiny system. As an extreme example^7^, the semiconductor films of 2.5 nm layers give observable pink noise. A small semiconductor can have pink noise down to $10^{-7}\,\textrm{Hz}$^8^, and voltage fluctuations through a semiconductor show pink noise from about $1\,\textrm{Hz}$ to $10^{-6.3}\,\textrm{Hz}$^9^. These remarkable low frequencies sound almost impossible for ordinary small systems. In this context, if the Wiener-Khinchin theorem $S(\omega )=\int _{-\infty }^{\infty }d\tau \int _{-\infty }^{\infty }dt\langle x(t)x(t-\tau )\rangle e^{-2\pi i\omega \tau }$ were correct, then the strong low-frequency signal in $S(\omega )$ of the pink noise would necessarily indicate the non-vanishing long-time correlation $\langle x(t)x(t-\tau )\rangle$. Probably, the time average in this theorem cannot be physical: It may not be well-defined for a non-stationary time series and cannot be accurately evaluated for a finite range of data.
3. **Apparent no lower cutoff in the PSD** It is often discussed that the pink noise does not seem to have an explicit lower cutoff in the PSD determined by any physics governing the system. The system exhibiting pink noise may not be stationary. Therefore, it may be useless to have discussions based on the stationarity of the system.
4. **Independence from dissipation** Remarkably, the pink noise appears even in the Hamiltonian mean-field (HMF) model, which is a strictly conservative system^10^ and has nothing to do with dissipation. Thus the usual fluctuation-dissipation theorem of the type $\left\langle \delta x^{2}\right\rangle \propto RkT$ may not hold for the pink noise(*R* is the electric resistance and *kT* is the temperature).
5. **Square of the original signal** When deriving the pink noise, it is often the case that the original time sequence is squared before the PSD analysis. For example, in the case of music^11^, the sound wave data should always be squared for PSD; the authors claim that this squared data is loudness. Similarly, in the case of the HMF model^10^, the authors always take a square of the original variables to obtain the pink noise. In both cases, the original data before taking the square shows no pink noise. In the case of the electric current, this procedure is not manifest, although the seminal paper^1^ emphasizes the square of voltage $V^{2}$ for PSD.

From the above five clues, we speculate that the beats of many waves with accumulating frequencies may be the origin of 1/f noise. A simple superposition of two waves $\sin (\omega t+\lambda t)+\sin (\omega t-\lambda t)=2\cos (\lambda t)\sin (\omega t)$ with $\omega \gg \lambda >0$ has no low frequency component around $\lambda$ in the PSD. On the other hand, the square of the superposed wave above has a low-frequency signal, *i.e.,* the beats, around $2\lambda$ in its PSD. Incidentally, it is sometimes confusing that the sound wave beat is “audible” although the PSD of the original superposition of the two waves does not show the corresponding low-frequency signal.

The above argument reminds us of a typical musical instrument, the Theremin^12^, which uses the wave beat. By mixing the high-frequency signals of 1000 kHz and 999.560 kHz generated by an electric circuit, the low-frequency signal of 440 Hz can be extracted as audible sound. The latter frequency can be varied slightly by the player’s hand, antenna distance, and capacitance to produce the desired frequency signal. Thus amplitude modulation can produce arbitrary low-frequency signals within a small-size system. The modulated signal has no intrinsic memory and has nothing to do with dissipation.

Another familiar device is the AM radio which clearly shows the wave beat or amplitude modulation (AM). By using 526.5 kHz to 1606.5 kHz radio waves, the low-frequency audible signal is extracted. In this case, the rectification (demodulation) process is essential to obtain audible low-frequency signals. This demodulation process is also essential for the pink noise in our proposal. In later sections, we will see a variety of pink noises in the many ways to demodulate.

The above five points will also be an elementary verification of our proposal. This will be discussed in later sections.

There appear to be several causes of the wave beat that forms pink noise, but the concentration of the wave frequencies is the essence of low-frequency signals. We will now focus on such causes separately in the following sections: (a) synchronization, (b) resonance, and (c) infrared divergence.

## Beats from synchronization

In this section, we will analyze the cause of wave beats, especially when the frequencies of the waves spontaneously approach with each other. We consider cooperative systems that exhibit this behavior.

### Exponential approach

The most typical type of synchronization would be the exponential approach, such as in the case of the Kuramoto model^13^, $\omega =e^{-\lambda t}$ where $\omega$ is the frequency and $\lambda$ is the approach speed, and *t* is the time. Then the frequency distribution function $P(\omega )$ and the time distribution function *p*(*t*) are related to each other by $P(\omega )d\omega =p(t)dt$. If we assume the stationarity of the fluctuation, we set $p(t)\equiv p=const$. Then,

1 $$\begin{aligned} P(\omega )=p(t)|d\omega /dt|^{-1}=p\lambda ^{-1}\omega ^{-1}5\omega ^{-1}. \end{aligned}$$

It is interesting that the exponential function gives the power index exactly $-1$.

**Figure 1:**
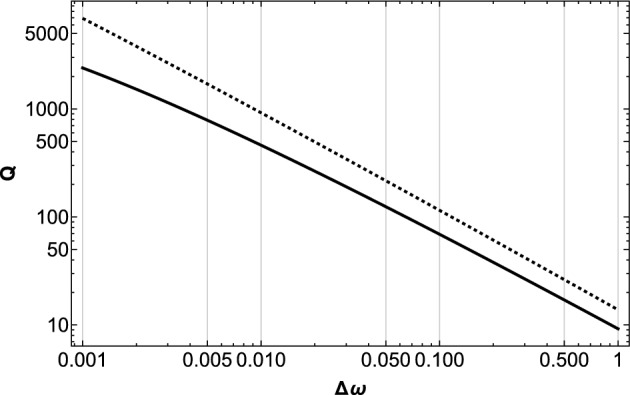
Examples of $Q(\Delta \omega )$ in Eq. (2) for the cases $p=1,\lambda =1,\omega _{2}=10^{5}$ and $\omega _{1}=10^{-4},10^{-6}$ (solid and dashed curves, respectively). The detailed behavior of $Q(\Delta \omega )$ depends on the upper and lower bounds of the integration.

The observed beat is the interference of the pair of frequency distributions above, and the beat frequency $\Delta \omega$ has its probability distribution function $Q(\Delta \omega )$ as

2 $$\begin{aligned} \begin{aligned} Q(\Delta \omega )&=\int _{\omega _{1}}^{\omega _{2}}d\omega P(\omega +\Delta \omega )P(\omega )\\&=\frac{p^{2}}{\lambda ^{2}\Delta \omega }\ln \left[ \frac{\omega _{2}\left( \omega _{1} +\Delta \omega \right) }{\omega _{1}\left( \omega _{2}+\Delta \omega \right) }\right] \\ \end{aligned} \end{aligned}$$

which again is proportional to $\left( \Delta \omega \right) ^{-1}$with small modification factor of $\ln [...\Delta \omega ]$. The detail of the full form $Q(\Delta \omega )$ depends on the boundaries of the integration domain $\omega _{1}<\Delta \omega <\omega _{2}$. Typical examples are shown in Fig. 1.

**Figure 2:**
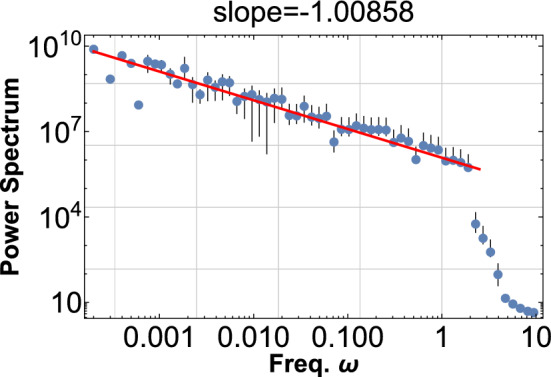
The PSD of $\phi ^{2}$ is shown with $\omega =10$, $c=0.2$, and *r* is a random field in the range [0, 30]. One thousand sine waves are superimposed according to Eq. (3). The power index can change up to about 0.1 for each run. This PDF shows the pink noise of index $-1$ for four decades.

The pink noise is robust, and the frequency distribution is directly reflected in the PDF of the waves at those frequencies,

3 $$\begin{aligned} \phi \left( t\right) =\sum _{i}\sin \left( 2\pi \omega (1+ce^{-r_{i}})t\right) , \end{aligned}$$

where $\omega$ is a fiducial frequency, *c* is a mixing constant, $r_{i}$ the Poisson random variable in some range for each sinusoid, and *i* runs from 1 to some upper limit. This general model, though static, represents the superposition of accumulating frequencies, including many dynamical systems. This is demonstrated in Fig. 2 where the PSD of $\phi ^{2}$ is shown.

**Figure 3:**
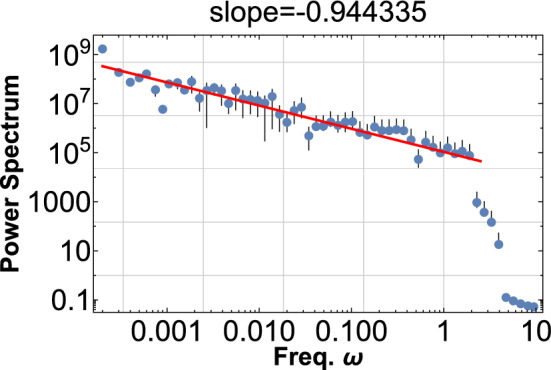
Same as Fig. 2, but the sine waves are superimposed with random phase $\theta _{i}$, $\,(0\le \theta _{i}<2{\pi })$ and the random amplitude $a_{i}\,(0\le a_{i}\le 1)$ for each: $\phi \left( t\right) =\sum _{i}a_{i}\sin \left( 2\pi \omega (1+ce^{-r_{i}})t+\theta _{i}\right)$. The power index drops a bit to $-0.9$, but this PDF shows the robustness of the pink noise from the wave beat.

The pink noise is robust, and the randomization of each phase of the sin-wave does not change the PDF except that the power index is slightly reduced, as shown in Fig. 3.

**Figure 4:**
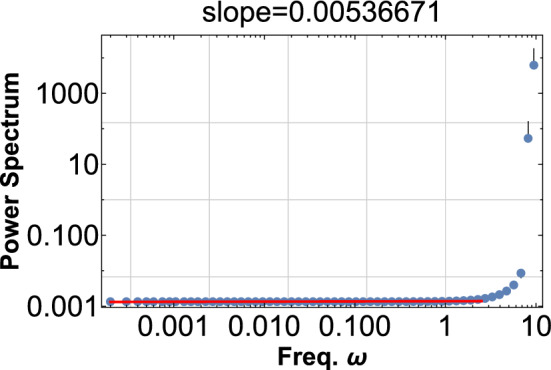
Same as Fig. 2, but this is PDF for the original signal $\phi$. Pink noise never appears in this case, indicating that the noise arises from the wave beat.

It is essential that the square of the signal $\phi ^{2}$ does show pink noise in PSD as in Fig. 1 while the original signal itself $\phi$ does not show any feature at low-frequency region as shown in Fig. 4. This fact manifestly demonstrates the pink noise comes from the wave beat.

### Power approach

Another popular type of synchronization would be the power approach $\omega =t^{-\alpha }$. Repeating the same calculations as above, we obtain the frequency distribution function as

4 $$\begin{aligned} P(\omega )=\underbrace{p(t)}_{p\text { const. }}|d\omega /dt|^{-1}=c\omega ^{-\beta } \end{aligned}$$

where $c\equiv p\alpha ^{-1},\beta \equiv \left( 1+\frac{1}{\alpha }\right) .$ The probability distribution function $Q(\Delta w)$ of the beat frequency $Q(\Delta w)$ is given by

5 $$\begin{aligned} Q(\Delta \omega )=\int _{\omega _{1}}^{\omega _{2}}d\omega P(\omega +\Delta \omega )P(\omega ) \end{aligned}$$

**Figure 5:**
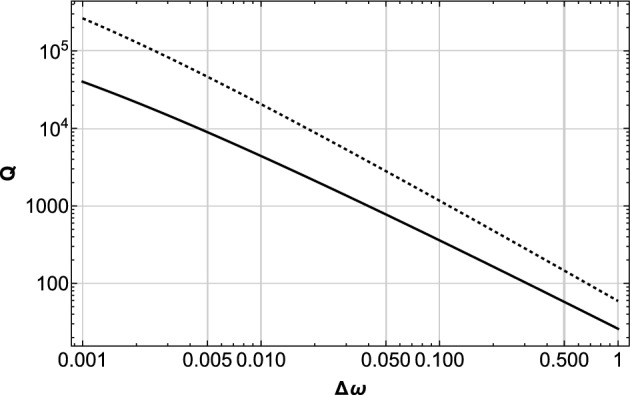
Examples of $Q(\Delta \omega )$ for the cases $p=1,\lambda =1,\omega _{2}=10^{5}$ and $\omega _{1}=10^{-4},c=1,\beta =1.2$ and 1.33 (solid and dashed curves, respectively).

Then,

6 $$\begin{aligned} Q(\Delta \omega )&=\frac{1}{\Delta \omega \left( 1-\beta \right) }\nonumber \\&\quad \left[ c^{2}\omega {}^{1-\beta }(\Delta \omega +\omega )^{1-\beta }{}_{2}F_{1}\left( 1,2-2\beta ;2-\beta ; -\frac{\omega }{\Delta \omega }\right) \right] _{\omega _{1}}^{\omega _{2}}\nonumber \\&\propto \Delta \omega {}^{-1-\left( 2/\alpha \right) }, \end{aligned}$$

if we expand with respect to small $\omega _{1}$ and small $\Delta \omega .$ The exponent is less than $-1$ for $\alpha >0$, and greater than $-1$ for $\alpha <0$ but the fiducial power is $-1$. Typical examples are shown in Fig. 5.

**Figure 6:**
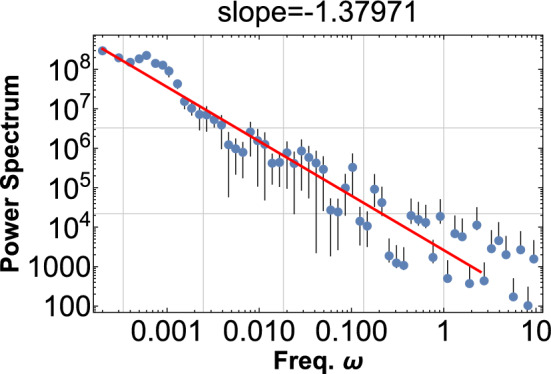
The PSD is shown for $\phi ^{2}$ with $\alpha =3,{\omega =10}$, $c=0.3$, and $r_{i}$ is a random field in the range [0,20]. One hundred sine waves are superimposed according to Eq. (7). This PDF shows the pink noise of index $-1.4$ for four decades.

**Figure 7:**
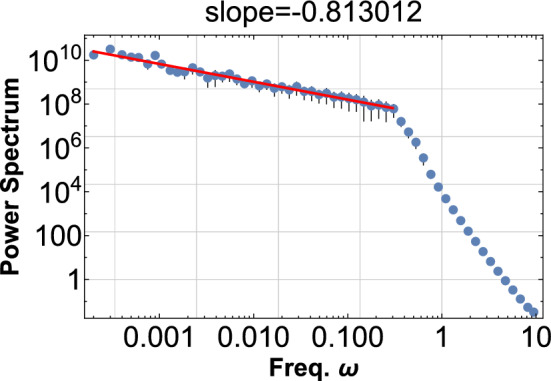
The PSD is shown for $\phi ^{2}$ with $\alpha =-3, {\omega =10},$ ${c=0.03}$, and $r_{i}$ is a random field in the range [0,1]. One thousand sine waves are superimposed according to Eq. (7). This PDF shows the pink noise of index $-0.8$ for three decades.

A typical wave signal can be constructed as before,

7 $$\begin{aligned} \phi =\sum _{i}\sin \left( 2\pi \omega (1+cr_{i}^{-\alpha })t\right) , \end{aligned}$$

and PSD for $\phi ^{2}$ are shown in Fig. 6 for $\alpha =3$, and in Fig. 7 for $\alpha =-3.$

Although the above demonstrations are typical simple models of the waves with accumulating frequencies, the frequencies are fixed. However, it is also possible to consider dynamical cooperative systems with time-dependent frequencies, and they often show pink noise; macroscopic coupled spin models^14^ and the Hamiltonian mean-field model^10^. Since the discussion of these is beyond the scope of this paper, we will cover them in a separate paper soon.

## Beats from resonance

We now consider the resonance, which produces the spontaneous concentration of frequencies and the wave beats. When the system with the intrinsic eigenfrequency $\Omega$ is stimulated (repeatedly), it emits the wave mode of the frequency $\Omega$ as well as those close to $\Omega$. Resonance thus ensures the concentration of frequencies in a small range. Since these frequencies are close to each other, the waves of these frequencies beat and produce a signal in low-frequency regions.

Suppose a typical case of the resonance characterized by the resonance curve, the Cauchy distribution

8 $$\begin{aligned} R[\omega ]=\frac{1}{\left( \frac{\kappa }{2}\right) ^{2}+\left( \omega -\Omega \right) {}^{2}}, \end{aligned}$$

where $\Omega$ is the resonance frequency and $\kappa$ characterizes the sharpness of the resonance. We will interpret this function $R[\omega ]$ as proportional to the number of $\omega$-modes in the resonator. Then the frequency distribution function $P(\omega )$ is given by the inverse function of $R[\omega ]$, as

9 $$\begin{aligned} \omega =R^{-1}[t]=\frac{\sqrt{-t\left( \kappa ^{2}t-4\right) }}{2t}+\Omega , \end{aligned}$$

where we have chosen the upper half of the inverse of $R[\omega ]$, since the lower half is symmetric to the upper half.

**Figure 8:**
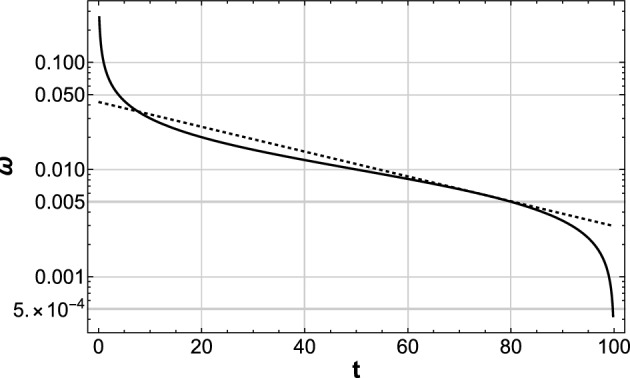
Demonstration of Eq. (9) in the log-linear graph. The function $\omega (t)$ can be approximated by the exponential function (dotted straight line) with the same inclination at the inflection point of $\omega (t)$, especially in the large-t range that is relevant for the low-frequency beats.

It is possible to make a naive approximation of Eq. (9) by the exponential function $\omega =Ae^{-Bt}$, where the constants *A*, *B* are determined at the inflection point of Eq. (9), as shown in Fig. 8. We already know that this exponential function gives the exact pink noise of slope $-1$ in PSD.

**Figure 9:**
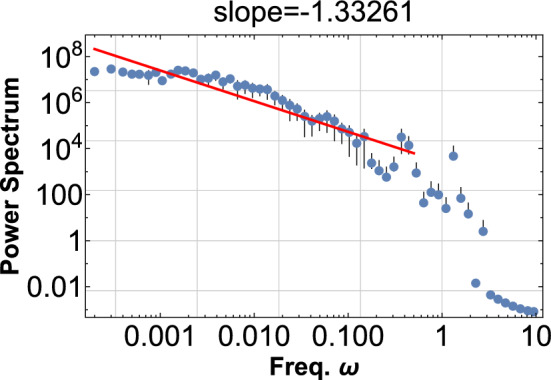
PSD of the time sequence $\phi (t)^{2}$ generated by Eq. (10) with $\kappa =0.1,\Omega =10,$ and the domain of the random field $r_{i}$ is [0, 10]. We have superimposed 100 sine waves, and this PSD shows the approximate power law of the index $-1.3$.

This is demonstrated in Fig. 9, where the PSD is plotted for the square $\phi (t)^{2}$of the time sequence $\phi (t)$ generated by

10 $$\begin{aligned} \phi (t)=\sum _{i}\sin \left( 2\pi R^{-1}\left( r_{i}\right) t\right) . \end{aligned}$$

However, the system analysis is not easy. Using the relation $P(\omega )d\omega =p(t)dt$ with $p(t)\equiv p=const$, we obtain the frequency distribution function $P(\omega )$ as

11 $$\begin{aligned} P(\omega )=p|d\omega /dt|^{-1}=\frac{32p(\omega -\Omega )}{\left( \kappa ^{2}+4(\omega -\Omega )^{2}\right) ^{2}}, \end{aligned}$$

which cannot be reduced to a single power form if $\kappa$ is finite.

Further complications arise from the actual resonant system, which has complicated overtones and multiple eigenfrequencies that systematically contribute to the pink noise. A fully systematic derivation of pink noise for each concrete resonant system requires further investigation. Since this is beyond the scope of this paper, we do not discuss it further here, but it will be analyzed in a separate paper soon.

## Beats from IR divergence

We now consider the third cause of the spontaneous concentration of frequencies from the infrared divergence. This class of systems exhibiting pink noise is quite diverse but can be reduced to the system composed of electrons and photons described by electrodynamics.

In this context, a quantum origin of pink noise was once proposed by using quantum interference of an electron between its pre- and post-scattering states^15,16^. They claim that the post-scattered electron state, after emission of a photon of frequency $\omega$, and the pre-scattered electron state interfere with each other to produce a beat of frequency $\omega$. However, this theory has been criticized^17,18^, mainly because quantum interference does not really occur; the pre- and post-scattered states are orthogonal with each other and have no chance to interfere. Even the introduction of the coherent state basis does not work. Incidentally, some other criticisms are not valid.

The essence of the pink noise may not be the self-interference of an electron, but the phase modulation of more macroscopic semi-classical objects associated with the emission of massless particles. In this section, we focus on such a semi-classical description of electromagnetism.

In the semiconductor, the electrons can be classical beyond the scale of the free streaming length, about 10 nm, which is several tends of the lattice size. When the system size is about 1 mm, there are $10^{10}$ such local coherent elements in the system. The electrons in such a coherent element can be described in terms of the wave packet,

12 $$\begin{aligned} \psi (x,t)=\textrm{e}^{i\left( k_{0}x-\omega _{0}t\right) }\int \phi \left( k_{0} +k^{\prime }\right) \exp \left[ ik^{\prime }\left( x-v_{g}t\right) \right] dk^{\prime } \end{aligned}$$

where $\phi \left( k\right)$ represents the weight function and $v_{g}=d\omega /dk$ is the group velocity^19^. The center of the wave packet represents the classical limit of the electron motion, and the expanse of the wave packet can represent interference.

When the wave packet of electrons meets impurity, the emission of photons changes its frequency by the emitted energy. The photon emission probability of energy $\hbar \omega$ is proportional to $\omega ^{-1}$and the frequency modulation of the wave packet amounts to $\omega$: this is the infrared divergence in the quantum electrodynamics (QED)^20^.

These wave packets in the system propagate in the same direction and cascade, bifurcate, and coalesce; the frequency modulation mixes up with each other along their propagation. We assume that the fiducial frequency of the wave packet is $\omega _{0}$, which is determined by the applied voltage and the conductivity. Then the original wave packets transform into the superposition of an enormous number of local packets with frequencies $\omega _{0}-\omega _{i},i=1,2,\ldots$. Each pair of these packets makes beats with all the possible differences $\left( \omega _{0}-\omega _{i}\right) -\left( \omega _{0}-\omega _{j}\right) =\omega _{j}-\omega _{i}.$ The system is thus filled with enormous number *N* of local wave packets $\psi _{i}(x,t)$, $i=1,2,\ldots \, N$. The total electric current is the superposition of all of them

13 $$\begin{aligned} \overrightarrow{J}=\frac{e}{2im}\sum _{i=1}^{N}\left\langle \psi _{i}(x,t)^{\dagger }\overrightarrow{\nabla }\psi _{i}(x,t)-\psi _{i}(x,t)\overrightarrow{\nabla }\psi _{i}(x,t)^{\dagger }\right\rangle , \end{aligned}$$

where the quadratic form of the wave packets corresponds to the demodulation of each packet, and thus the pink noise appears in this current. This process is the same as the previous case of the exponential approach, and many wave packets with slightly different frequencies interfere to give the wave beat as in Eq. (2) and thus the pink noise appears as in Fig. 2.

It is important to notice that the full quantum interference, including the emitted photons, is not needed for generating pink noise, but a tremendous number of wave packets with synchronized waves are crucial. The emitted photons are easily absorbed in the system, and therefore the Faraday cage surrounding the system, if any, does not affect the current fluctuations at all^18^.

In this context, the coherent dressed state formalism for QED was developed to cancel the infrared divergence associated with the massless photon^21,22^. Although most authors assume (semi-)classical background currents ab initio, the classical degrees of freedom are not correctly derived. The derivation of the classical degrees of freedom in QED is possible in the closed time-path formalism of the effective action associated with an unstable state. The IR divergence of the theory requires the separation of the classical statistical kernel from the complex effective action. Then the Langevin equation with classical noise is derived from the effective action and can describe the classical evolution of currents^23^.

This formalism requires a more systematic discussion than we can give here. However, we will report this theory in a separate paper, including the classical-quantum interference.

## Discussions

So far, we have proposed three kinds of origin of the synchronizing waves, which give systematic beats and produce pink noise. Since the pink noise is generated by the wave beat or the amplitude modulation, any demodulation process is required for observation. This demodulation process may be (a) intrinsic mechanisms associated with the system or (b) operational processes associated with the data reduction for PSD. In either case, the demodulation process provides robustness and a variety of pink noise. This section is devoted to showing some examples of such robustness and variety.

1. *Fiducial* The fiducial signal is the one discussed in "Exponential approach", with the same parameters of Fig. 2: $\omega =10$, $c=0.2$, and $r_{i}$ is a random field in the range [0, 30]. There, $10^{3}$ sinusoids are superimposed according to Eq. (7). The squared signal $\phi ^{2}$shows a clear pink noise of slope $-1.0$ as in Fig. 2.
2. *The threshold for* $\phi ^{2}$ We set the new data zero for the $\phi ^{2}$ data that is smaller than the mean and leave the other data as they are. The PSD shows pink noise with a slope of $-1.0$, almost unchanged from the fiducial case. This case may apply to the nerve system, where only a voltage greater than some threshold can produce a spike signal.
3. *On–off threshold for* $\phi ^{2}$ We set the new data zero for the $\phi ^{2}$ data that is less than the mean and set the other data to 1. The PSD shows pink noise with a slope of $-0.94$.
4. *On–off inverse threshold for* $\phi ^{2}$ This is the opposite of case 3. We set the value 1 for the $\phi ^{2}$ data that is smaller than the mean and set the other $\phi ^{2}$data to 0. The PSD shows pink noise with a slope of $-0.94$, the same as in case 3.
5. *Threshold for original data* $\phi$ we set the new data zero for the $\phi$ data smaller than the mean and set the other data as is. The PSD shows pink noise with a slope of $-0.98$.
6. *Rectification of the original data* $\phi$ We set the new data to zero for the $\phi$ data that is negative and leave the other data as is. The PSD shows pink noise with a slope of $-1.2$. This may apply to some electric circuits containing transistors, diodes, and vacuum tubes.
7. *Sequence of locally averaged* $\phi ^{2}$ We divide the entire time sequence of $\phi$ into $10^{3}$ segments and apply a quadratic average in each segment. The PSD shows pink noise with a slope of $-1.1$. This is the data treatment in the original experiment^1^.
8. *Sequence of locally averaged* $\phi$ Same as case 7, but we apply a simple average in each segment. The PSD shows NO pink noise, and the power is positive $+0.8$.
9. *Coarse time resolution for* $\phi ^{2}$ We reduce the number of sample points to half of the original. The PSD shows an almost pink noise with a slope of $-1.1$.
10. *Fewer superimposed waves* We reduce the number of superimposed waves from the fiducial $10^{3}$ to 10. The PSD shows NO pink noise.
11. *More superimposed waves* We increase the number of superimposed waves from the fiducial $10^{3}$ to $10^{4}$. The PSD shows pink noise with a power of $-0.94$.
12. *Longer time sequence* We extend the time sequence from the fiducial $10^{4}$ to $10^{5}$. The PSD shows pink noise with a slope of $-1.0$; the same as before, but with a power law extended by a decade.
13. *Multiple fiducial frequencies* We changed the fiducial frequency from the original single to 5, randomly selected from 0 to 20. The PSD shows pink noise with a slope of $-1.5$.

As examined above, there are multiple demodulation processes. They are classified as (a) system-intrinsic and (b) operational in the data reduction, although the classification is not exclusive. Examples of (a) are thresholding and rectification: cases 3,4,5,6. Examples of (b) are data squaring: cases 1, 2, 7. Cases 9, 11, 12, 13 show some robustness of pink noise.

We have considered pink noise widely by defining the noise with power-law of the index $-\alpha$, ($0.5<\alpha <1.5$) and studied a model which shows this behavior. However, there is a class of systems that shows exactly the power $-1$. Our model cannot explain this exact power $-1$, except the exponential approach in Sect. "Exponential approach". We would like to explore how extent the exponential approach model can be general in the future.

## Conclusions and prospects

We have discussed the origin of pink noise from the beat of waves with accumulating frequencies. We have examined three possible causes for this cooperative effect: synchronization, resonance, and IR divergence. There may be more mechanisms. We point out the verifiability/falsifiability of our model based on the five crucial observations for the pink noise in Sect. "Method: some crucial clues for pink noise".

1. **Wave** The wave is essential for producing beat and amplitude modulation. The wave may be hidden inside the system, and the data may be obtained after it passes through the threshold. If we cannot find a coherent wave in the system, our model cannot be applied.
2. **Small system and apparent long memory** The Wiener-Khinchin theorem, when applied to the pink noise, may indicate an extremely long memory. However, according to our model, this long memory is not indispensable. Our model will not be essential if we find real long memory in the system that shows pink noise.
3. **Apparent no lower cutoff in the PSD** The beat of the waves with accumulating frequencies or the amplitude modulation can yield an infinitely low-frequency signal from inside a finite system within the observational constraints. Therefore, if an intrinsic lower-cutoff frequency is found in the pink noise, our model cannot be applied.
4. **Independence from dissipation** The beat of the wave or the amplitude modulation is a secondary fluctuation caused by wave synthesis. Therefore, the dissipation may destroy the pink noise because it may cancel the fragile wave beats.
5. **Square of the original signal (necessity of the demodulation process)** The amplitude modulation needs some demodulation process for observation. The primary fluctuations before the demodulation do not appear in the PSD. Our model for pink noise predicts the demodulation process as either (a) intrinsic to the system or (b) operational in the data reduction. If the demodulation is found in the system of pink noise, and the pink noise disappears when the demodulation process is removed, then our model is strongly favored.

Although we have proposed a basic model of pink noise, we still have many problems with elaborating on the present formalism. Some have already been described in appropriate places with the keyword ’separate paper’. They are dynamical cooperative systems, actual resonant systems, and systems with IR divergence. Among them, we summarize the possibly resonant systems in Table 1.

**Table 1:** List of systems that show pink noise possibly due to the resonance effect, as we have discussed in "Beats from resonance". This Table is preliminary, and the final analysis will be reported in our papers soon.

	System	Resonant mode	Demodulation	Description
1	Earthquakes	Earth free oscillation^24^	Fault rupture	USGS World 50 years data, magnitude 4-5 show pink noise PSD of slope $-1.2$ below 2.5 months.
2	Icequakes	Iceberg eigenfrequency	Ice fault rupture	NOAA Icequakes (Bloop) deep-sea sound^25^ show pink noise with slope $-0.8$.
3	Solar flare	5 min oscillation^26^	Magnetic reconnection	HESSI^27^ solar flare luminosity curve for 16 years shows pink noise with slope $-0.9$
4	Sunspots	Same as above or macro spin model^14^	(intrinsic)	Sunspot number time sequence from the year 1820 to 2010 shows pink noise of slope $-1.1$ over the entire period^14^.
5	$NO_{3}^{-}$	Same as above	(intrinsic)	$NO_{3}^{-}$—Concentration during the years 1610–1904 in the DF01 antactica ice core^28^ shows pink noise with slope $-1.1$.
6	Variable stars	Same as above	(intrinsic)	Some of the variable stars show pink noise. The light courve of Mira (Red giant) for about 6 years^29^ shows pink noise with slope $-1.2$.
7	Suikinkutsu	2 m pottery cavity underground	Data squared	The water harp cave at HosenIn Kyoto shows pink noise of slope $-0.8$(left) and $-0.6$(right) for about four decades.
8	Big gong	Eigenfrequency	Data squared	The big gong in Enkohji-Temple Kyoto shows pink noise with slope $-1.6$. The small gong shows NO pink noise.

The list in Table 1 is tentative and imperfect. It will be completed in our future publications, including the verification of our simple pink noise model.

## Data Availability

The datasets used and/or analysed during the current study available from the corresponding author on reasonable request.
